# RNAi-Mediated Downregulation of Inositol Pentakisphosphate Kinase (*IPK1*) in Wheat Grains Decreases Phytic Acid Levels and Increases Fe and Zn Accumulation

**DOI:** 10.3389/fpls.2018.00259

**Published:** 2018-03-06

**Authors:** Sipla Aggarwal, Anil Kumar, Kaushal K. Bhati, Gazaldeep Kaur, Vishnu Shukla, Siddharth Tiwari, Ajay K. Pandey

**Affiliations:** ^1^Department of Biotechnology, National Agri-Food Biotechnology Institute, Mohali, India; ^2^Department of Biotechnology, Panjab University, Chandigarh, India; ^3^Copenhagen Plant Science Centre, PLEN, University of Copenhagen, Copenhagen, Denmark

**Keywords:** phytic acid, *Triticum aestivum*, inositol penta*kis*phosphate kinase, gene silencing, wheat transformation

## Abstract

Enhancement of micronutrient bioavailability is crucial to address the malnutrition in the developing countries. Various approaches employed to address the micronutrient bioavailability are showing promising signs, especially in cereal crops. Phytic acid (PA) is considered as a major antinutrient due to its ability to chelate important micronutrients and thereby restricting their bioavailability. Therefore, manipulating PA biosynthesis pathway has largely been explored to overcome the pleiotropic effect in different crop species. Recently, we reported that functional wheat inositol penta*kis*phosphate kinase (*TaIPK1*) is involved in PA biosynthesis, however, the functional roles of the *IPK1* gene in wheat remains elusive. In this study, RNAi-mediated gene silencing was performed for *IPK1* transcripts in hexaploid wheat. Four non-segregating RNAi lines of wheat were selected for detailed study (S3-D-6-1; S6-K-3-3; S6-K-6-10 and S16-D-9-5). Homozygous transgenic RNAi lines at T_4_ seeds with a decreased transcript of *TaIPK1* showed 28–56% reduction of the PA. Silencing of *IPK1* also resulted in increased free phosphate in mature grains. Although, no phenotypic changes in the spike was observed but, lowering of grain PA resulted in the reduced number of seeds per spikelet. The lowering of grain PA was also accompanied by a significant increase in iron (Fe) and zinc (Zn) content, thereby enhancing their molar ratios (Zn:PA and Fe:PA). Overall, this work suggests that *IPK1* is a promising candidate for employing genome editing tools to address the mineral accumulation in wheat grains.

## Introduction

Mineral malnutrition is among the most critical worldwide challenges to humankind. In developing countries ∼50% of the children are estimated to be micronutrient deficient (WHO, 2007). This condition might be a result of crop production in areas with low mineral phytoavailability and/or consumption of staple crops with inherently low tissue mineral concentrations (Welch and Graham, 2005). The mineral malnutrition can be addressed through dietary enhancement, mineral supplementation, food fortification, or increasing the concentrations and/or bioavailability of mineral elements. However, methodologies to expand dietary enhancement, mineral supplementation, and food fortification have not generally been very effective (White and Broadley, 2005). Thus, biofortification of crops by decreasing the concentration of antinutrients and other secondary metabolites can be considered as an important means to increase the bioavailability of mineral elements.

Cereal crops are rich sources of mineral ions, but the presence of certain anti-nutritional components limits their bioavailability (Raboy, 2009). Phytic acid (*myo*-inositol hexa*kis*phosphate, PA, IP_6_) is one of the major anti-nutritional component in grains of wheat, rice, soybean, and other crops, that limit the bioavailability of micronutrients. In grains PA is major storage form of phosphorus (P). The developing grains accumulate PA in vacuoles as phytate, that chelates important micronutrients and thereby causing drastic reduction in the bioavailability of mineral ions (Garcìa-Estepa et al., 1999). Additionally, PA cannot be utilized efficiently by monogastric animals due to lack of phytase enzyme. Furthermore, excretion of undigested PA in animal waste contributes to environmental phosphate pollution, by accelerating soil eutrophication (Stevenson-Paulik et al., 2005). Therefore, reducing the PA content in cereal grains is a desired goal for the genetic improvement of several crops to address the dual issues of micronutrient bioavailability and eutrophication (Raboy, 2009; Secco et al., 2017).

Phytic acid biosynthesis pathway genes have been reported from multiple crop species (Stevenson-Paulik et al., 2002; Suzuki et al., 2007; Sweetman et al., 2007; Stiles et al., 2008). PA in plants can be synthesized by a lipid dependent or independent pathway depending on the primary inositol source (Stevenson-Paulik et al., 2005; Raboy, 2009). Lipid-independent pathway is predominant in seeds of cereals and legumes. The first committed step performed by *myo*-inositol-3-phosphate synthase (MIPS) involves the formation of inositol-3-phosphate (Ins3P) from glucose-6-phosphate. Subsequent steps involves sequential and ordered phosphorylation at the remaining five positions of the inositol ring through various enzymes (Stiles et al., 2008; Rasmussen et al., 2010). The enzymes catalyzing these phosphorylation reactions include inositol monophosphatase (IMP), inositol tris/tetraphosphate kinase (ITPK), inositol polyphosphate kinase (IPK2) and inositol-penta*kis*phosphate 2-kinase (IPK1) (Raboy, 2009; Rasmussen et al., 2010).

Based on the physiological properties, low phytic acid (*lpa*) mutants in maize have been characterized as *lpa1*, *lpa2* or *lpa3*. Maize *lpa1* mutant was shown to be defective in a multi-drug resistance-associated protein (MRP) ABC transporter with unknown function (Shi et al., 2007). Maize *lpa2* mutant is caused by mutation in an inositol phosphate kinase gene (Shi et al., 2003). The maize *lpa3* mutant had mutation in a gene that encodes *myo*-inositol kinase enzyme (Shi et al., 2005). Barley *lpa2* mutant lines (M635 and M955) had enhanced free phosphate (Pi) and decreased PA levels due to loss of inositol phosphate kinase activity (Dorsch et al., 2003). Utilizing reverse genetics in *Arabidopsis* led to the identification of genes that are necessary for PA accumulation in seed (Kim and Tai, 2011). These observations indicated that most of the PA pathway genes could be functionally conserved among multiple crops.

The hexaploid wheat (*Triticum aestivum* L.), is one of the most important staple food crop providing nearly 55% of the carbohydrates and 20% of the food calories consumed globally (Breiman and Graur, 1995). The initial lack of information about PA biosynthesis pathway genes from wheat and inefficient genetic transformation were limiting factors hindering the PA associated research. To address this issue, we reported PA biosynthesis pathway genes and putative PA transporter from hexaploid wheat that suggested to be potential candidates for gene suppression (Bhati et al., 2014; Aggarwal et al., 2015). Further silencing of wheat *ABCC13*, a putative transporter of PA resulted in reduced PA without significantly affecting the levels of zinc (Zn) and iron (Fe) in the grains. Moreover, other morphological changes suggest a multifunctional aspect of this transporter (Bhati et al., 2016). The pleiotropic effects and compromised spike development in *TaABCC13* silenced plants encouraged to explore alternative approaches for achieving low PA. Studies have indicated that genes involved during the early and late biosynthesis pathway could be the potential targets for achieving low PA (Raboy, 2009; Rasmussen et al., 2010). This has been successfully demonstrated by targeting genes related to PA biosynthesis in barley (Ye et al., 2011), maize (Shi et al., 2007), *Arabidopsis* (Stevenson-Paulik et al., 2005) and rice (Ali et al., 2013a,b). Although enhanced micronutrient content was reported in these low PA crops, no molecular basis was accounted for such trait.

Previously, TaIPK1 was shown to be a functionally active that is differentially expressed at the transcript level and putatively catalyzes the last step of PA biosynthesis (Bhati et al., 2014). Transcript abundance of *TaIPK1* derived from either B or D genomes were highly expressed when compared to the A genome. Subsequently, *IPK1* was targeted for RNAi-mediated gene silencing and its effects were studied in hexaploid wheat (C306). Multiple non-segregating RNAi lines (S3-D-6-1; S6-K-3-3; S6-K-6-10 and S16-D-9-5) were selected for detailed characterization of the *IPK1* transcript reduction effect. Silencing of *IPK1* resulted in significant lowering of grain PA along with increase in levels of free Pi. The decrease of phytate in wheat grains was accompanied by the increase in the content of micronutrients like Fe and Zn. In addition, lowering of the wheat *IPK1* transcripts also showed pleiotropic effects that includes decreased grain yield.

## Materials and Methods

### Plant Material and Growth Conditions

Hexaploid wheat (*Triticum aestivum*) variety, C306, was used for this study. For genetic transformation, surface sterilized seeds of C306 were sown in pots with sterile vermiculite and kept in a plant growth chamber (Conviron, Canada) under a 16 h photoperiod at 400 μmol⋅m^-2^⋅s^-1^, 70% relative humidity and 25°C/18°C (day/night). For wheat transformation tagged spikes were harvested at 12–16 DAA. To study gene expression, the main individual spikes of the transgenic wheat and respective controls were tagged at the first DAA. Subsequently seeds from the tagged spikes were harvested at 14 or 21 DAA in liquid nitrogen for RNA extraction.

### Designing of RNAi Constructs

Previously reported RNAi vector pMCG161 (TAIR stock-CD3-459) was used for the genetic transformation of wheat as mentioned earlier (Zalewski et al., 2010; Gasparis et al., 2011; Bhati et al., 2016). The T-DNA of the vector contained *bar* gene (also called pat, phosphinothricin acetyl transferase) as a selectable marker that confers resistance to herbicide BASTA and allows the selection of transgenic plants over the optimized concentration. RNAi cassette is expressed under the control of CaMV 35S promoter, while monocot specific UBI intron promoter controls expression of *bar* gene. Cloning of 320 bp fragment of *IPK1* in sense and anti-sense orientation was done in pMCG161 vector using two restriction sites separated by a rice waxy intron. Primers were designed from the conserved region of all the homoeologs, i.e., TaIPK1:2AL-TRIAE_CS42_ 2AL_TGACv1_095050_AA0306410, TaIPK1:2BL-TRIAE_CS42_ 2BL_TGACv1_129504_AA0386330, TaIPK1:2DL-TRIAE_ CS42_2DL_TGACv1_159606_AA0540500 (Supplementary Figure S1) with *AscI/AvrII* overhangs in forward primer and *SpeI/SgfI* overhangs in reverse primer. The sequences of primers were given in Supplementary Table S1. Restriction analysis using multiple enzymes and sequencing confirmed the cloning of sense and anti-sense sequences in silencing cassette. The confirmed RNAi construct was transformed into the AGL1 strain of *Agrobacterium tumefaciens* and subsequently used for wheat transformation.

### *Agrobacterium*-Mediated Wheat Transformation

A single clone of *Agrobacterium* confirmed for the presence of *TaIPK1*:pMCG161 RNAi construct was used for wheat transformation. Wheat transformation was performed as described earlier with minor modifications (Przetakiewicz et al., 2004; Gasparis et al., 2011; Bhati et al., 2016). In brief, seeds harvested from the 14–16 DAA tagged spikes were surface sterilized with NaClO (1.2% v/v in 10% ethyl alcohol). Approximately 700 immature (1 to 1.2 mm) embryos were isolated by dissecting the seeds aseptically and then cultured on callus induction media consisting of MS medium with 2 mg L^-1^ of 2,4-dichlorophenoxyacetic acid (2,4-D). The callus inducing plates carrying 25–30 immature embryos were incubated for 72 h at 22°C/dark. After incubation, embryos were infected with co-cultivation suspension of AGL1 strain (OD_600_ 0.3–0.5) harboring RNAi construct and the suspension containing Silwet (0.001% v/v) was spotted drop by drop on each growing calli (Gasparis et al., 2011). Co-cultivation step was followed by washing with autoclaved MilliQ water containing Cefotaxime (350 mg L^-1^). Washed calli were transferred onto the callus induction media having Cefotaxime (250 mg L^-1^). The plates were then incubated at 22°C/dark for three cycles of 12 days with sub culturing to fresh media after each cycle. Healthy calli developed on Post-infection callus induction media were then subjected to herbicide selection by subculturing it on 2 mg L^-1^ BASTA along with growth supplement zeatin, 1 mg L^-1^. These plates were then incubated at 22°C/light and sub-culturing was performed for three cycles of 12 days each. The developing plantlets were then further cultured on half- strength MS medium containing 2.5mg L^-1^ BASTA for two cycles. Later, plantlets with healthy rooting were transferred to vermiculite pots for hardening. The shoots regenerated from independent calli were considered as independent T-DNA integration events and assigned as T_0_ putative plants.

### Transgene Integration and Segregation Analysis

The plants those survived the hardening were checked for gene integration through PCR. Total genomic DNA was isolated from young leaves of putative transgenic and non-transformed (control) plants using the DNeasy Plant Mini Kit–QIAGEN following manufacturer protocol. Primers were designed for amplification of the *bar* gene and OCS region of RNAi cassette (Supplementary Table S1). Leaves from the individual tiller of each T_0_ plant were also screened by PCR to eliminate the possibility of chimeric plants. Amplicons generated from PCR-positive T_0_ plants were purified and sequenced for confirmation. Subsequently, seeds collected from the PCR-positive tillers of T_0_ plants were subjected to segregation analysis on 2.5 mg L^-1^ BASTA that was optimized for cultivar C306 (control wheat) in hydroponic media. These plantlets were transferred to soil pots, after collecting data for survival ratio. Further, the selection fidelity was confirmed with PCR by amplifying and sequencing of a *bar* fragment gene. Plants that are positive in PCR amplification and follows Mendelian segregation ratio were cultivated up to the T_2_ and T_3_ generations. For T_4_ generation, ten plants from three non-segregating independent transgenic lines (S3, S6, and S16) were propagated and used for further analysis.

### Gene Expression Analysis by Using Quantitative RT-PCR

For the confirmation of gene silencing, SYBR Green (Quanti-Tect SYBR Green RT-PCR Master mix, QIAGEN) based reactions were performed on ABI PRISM 7500 Fast Real-Time Platform (Applied Biosystems). The silencing of the gene was confirmed in the immature seeds of the developing grains at T_4_. During the wheat transformation wheat lines derived from callus showing no integration of the vector were subsequently propagated along with the transgenic wheat as a control plants for comparative analysis. The main individual spikes of selected T_3_ plants were tagged at the first DAA and tissue samples were harvested at 14 DAA in liquid nitrogen. RNA was extracted using the RNeasy Plant Mini Kit (Qiagen, Valencia, CA, United States), following manufacturer’s instructions. gDNA free cDNA was prepared using 2 μg of RNA. Transcriptor First Strand cDNA Synthesis Kit RT-PCR (Roche, Indianapolis, IN, United States) was used for cDNA preparation following the manufacturer’s guidelines. Further, level of silencing was checked using real time PCR primers targeting *TaIPK1* CDS region other than RNAi fragments. The sequences of primers are listed in Supplementary Table S1. Relative expression level was quantified using 2^-ΔΔCt^ method after normalizing *C*_t_ values against wheat ADP-ribosylation factor 1 (*ARF1*) and 18S rRNA genes as an internal control (Livak and Schmittgen, 2001).

### Morphological Analyses of Transgenic Plants

*TaIPK1*:RNAi lines cultivated in plant growth chamber were observed for spike characteristics that include spikelet count, spike length, and awn length. Spikes from each T_3_ line (only from primary tiller) that showed reduction in *TaIPK1* transcript level were considered for above observations along with their respective controls. The spikes of the transgenic wheat were observed for the head sterility under the light microscope (Leica 2500LCD). Seed count was done from the primary spikes of the C306 and RNAi lines. Average seed weight was measured by taking 50 random seeds from C306 and RNAi lines. Further, grain morphology was also observed for C306 and transgenic RNAi lines using light microscope.

### Germination Assays

The germination pattern of the *TaIPK1*:RNAi lines and non-transformed plants were observed to check any effect of the integration of the plasmid or transgenic events. Seeds were surface sterilized with NaClO (1.2% v/v in 10% ethyl alcohol). Seeds were then transferred on distilled water-soaked filter paper, kept at the base in Petri dishes. The plates were kept in a plant growth chamber maintained at 25°C/18°C (day/night) and 70% relative humidity. Pictures of seeds were taken after 48 and 72 h post-germination.

### PA and Inorganic Phosphate (Pi) Estimation

Total PA was estimated in mature seeds of T_3_ and T_4_ lines along with controls using K-PHYT kit (Megazyme Inc, Bray, Ireland) as per the manufacturer’s protocol. Briefly, the seeds were grounded to fine powder and extracted using 0.66 N HCl with continuous stirring overnight. The supernatant was then used for colorimetric development as mentioned in the manufacturer instruction booklet. For free phosphate estimation, mature seeds from selected *TaIPK1*:RNAi lines were used along with control plants. Free phosphate was measured using ascorbate and ammonium molybdate method (Ames, 1966).

### Micronutrient and Total Phosphorus (P) Analyses Using ICP-MS

Total seed phosphorus and metal analysis was performed using Inductive Coupled Plasma-MS (ICP-MS). Mature seeds of T_3_ and T_4_ lines along with control plants were grounded to fine powder and subjected to the microwave-digested with HNO_3_ (SuraPure^TM^, Merck). Subsequently, it was analyzed as described previously (Bhati et al., 2014). For analysis in T_4_ seeds, three to four plants of the respective transgenic lines were chosen for calculating an average accumulation of micronutrients.

### *In Silico* Analysis

The expression pattern for the *TaIPK1* homoeologs were extracted as transcripts per million (TPMs) from Wheat Expression Browser expVIP: http://www.wheat-expression.com/ (Borrill et al., 2015). Expression was performed during different developmental stages of grain, leaf, root, spike, and stem at the mentioned Zadoks scale (Zadoks et al., 1974). Five different grain tissues (whole endosperm, starchy endosperm, transfer cells, aleurone, and aleurone+endosperm) and three developmental stages consisting of 10, 20, and 30 DPA (days post-anthesis) for wheat was utilized to check the expression of *TaIPK1* homoeologs.

## Results

### Homeologous Dependent Expression Pattern of *TaIPK1*

**FIGURE 1:**
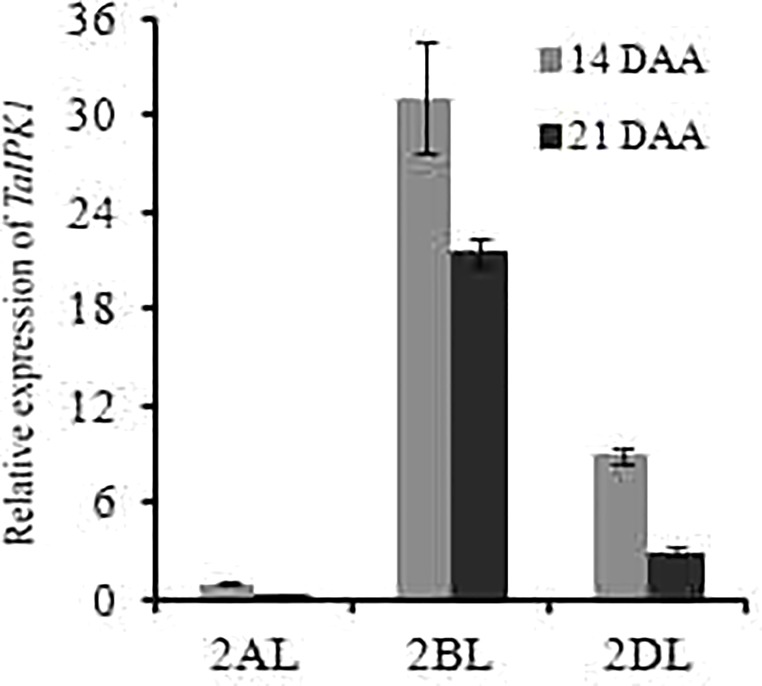
Differential expression analysis of three homoeologs of *TaIPK1* at two seed developmental stages (14 and 21 DAA). Transcript specific primers were designed for 2AL, 2BL, 2DL of *TaIPK1* homoeologs based on genomic information available at IWGSC. gDNA free cDNA was prepared using 2 μg of RNA. qRT-PCR assays were performed using SYBR green and *C*_t_ values were normalized against wheat ADP-ribosylation factor 1 (*ARF1*) as an internal control. The indicated error bars represents the standard deviation from three independent replicates.

The expression patterns for *IPK1* homoeolog genes was performed in wheat tissues, by using the Wheatexp; Wheat Expression Browser (Choulet et al., 2014; Pfeifer et al., 2014; Borrill et al., 2015). The expression for homoeolog-specific transcript of *TaIPK1* gene was determined in five different tissues, i.e., grain, leaf, root, spike, stem and at three developmental stages (Z71, Z75, and Z85). Our analysis suggested expression from all the three homoeologous alleles. A higher transcript level for *TaIPK1*-2B homoeolog was observed in most of the examined tissues (Supplementary Figure S2A). Furthermore, identical expression of *TaIPK1* homoeologs was observed in different grain tissues suggesting the importance of all the gene transcripts during grain development (Supplementary Figure S2B). The expression validation for *TaIPK1* homoeologs was performed in C306, an Indian hexaploid wheat variety. qRT-PCR showed high transcript level of *TaIPK1-2B* at different developmental stages (14 and 21 DAA) (**Figure 1**).

### Preparation of RNAi Construct, Wheat Transformation and Screening of T_0_ Transgenic Plants

**FIGURE 2:**
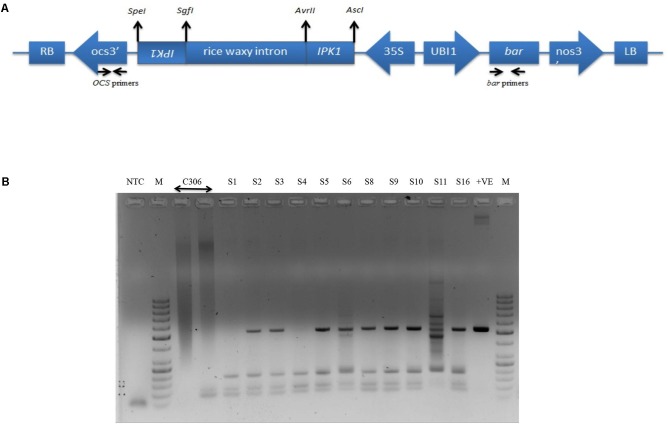
Schematic representation and confirmation of the RNAi construct in wheat for targeted gene silencing of *TaIPK1*. **(A)** Vector backbone of pMCG161 was utilized to clone fragments of *TaIPK1* gene in the sense and antisense orientations. *bar* gene was used as a plant selection marker **(B)** Representative picture for the screening of the putative transgenics to confirm the genomic integration of the RNAi constructs in wheat. PCR amplification of *bar* gene from the genomic DNA of 11 independent integration events (T_0_ stage) recovered after hardening procedure (S1–S6, S8–S11, S16 are putative transgenic plants, C306, control plant, +ve, plasmid pMCG161, NTC, no template control).

RNAi construct was designed to achieve reduced endogenous *TaIPK1* mRNA. A schematic representation of the RNAi construct is presented (**Figure 2A**). The *Agrobacterium* strain AGL1 expressing *TaIPK1*:pMCG161 RNAi construct was used for wheat transformation. A total of 16 plantlets originating from independent calli were obtained after herbicide (BASTA) selection (Supplementary Figures S3A,B). Only eleven plantlets survived during the hardening procedure in the soilrite containing pots. Since, these plantlets were derived from distinct callus, they were considered as independent transgenic events for pMCG161:*TaIPK1* RNAi T-DNA insertion. T_0_ plants surviving the hardening procedure in soil were named as S1–S6, S8–S11 and S16. Further, screening of these plants was performed by PCR amplification of *bar* and *OCS1* terminator sequences that resulted in the confirmation of T-DNA insertion in nine plantlets (**Figure 2B**). During various stages of screening, the PCR fragments were sequenced to confirm the presence of *bar* or *OCS1* sequences (Supplementary Figure S4). Three independent events (S3, S6, and S16) showed healthy growth under selection and screening procedures were chosen for further analysis. Progenies of these three events were propagated till the T_3_/T_4_ generation.

### Silencing of *TaIPK1* Enhances Free Pi and Decreases Total PA

**FIGURE 3:**
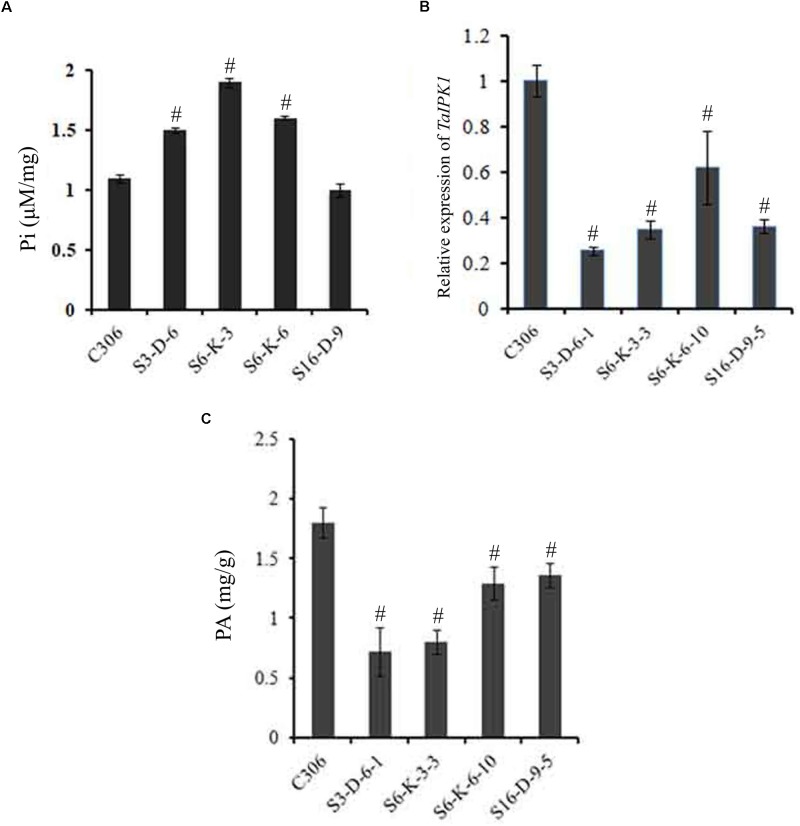
Confirmation of silencing in *TaIPK1*: RNAi lines in T_4_ seeds. **(A)** Free Pi content of control C306 and *TaIPK1*:RNAi lines were estimated using colorimetric based assays. **(B)** Relative fold change of *TaIPK1* expression in wheat transgenic lines. RNAi lines from three independent events were subjected to expression analysis at 14 DAA stage. The cDNA templates were prepared from 2 μg of DNase free RNA. qRT-PCR assays were performed using SYBR green and *C*_t_ values were normalized against wheat ADP-ribosylation factor 1 (*ARF1*) as an internal control. **(C)** Total phytic acid in mature wheat grains of transgenic lines (T_4_). PA was measured in the mature seeds collected from the primary tiller of each line. ^#^Indicates significant differences at *p* < 0.05.

The *lpa2* mutant phenotype is generally accompanied by enhanced accumulation of free Pi (Raboy, 2009; Yuan et al., 2012). Therefore, estimation for Pi was performed in the multiple lines of T_3_ seeds. Our analysis, showed a significant increase in the Pi level in most of the transgenic lines (S3-D-6, S6-K-3, and S6-K-6) as compared to control. In general, an increase in the transgenic grain Pi ranged from ∼1.2- to 1.7-fold, as compared to the control (**Figure 3A**). These lines were selected for further study.

The relative quantification of gene silencing was performed using qRT-PCR in the multiple lines of the T_4_ seeds (14 DAA). Expression data indicated consistent and significant decrease in the transcript levels of the *TaIPK1* in the T_4_ seeds as represented in Supplementary Figure S5. This suggests an effective silencing of wheat *IPK1* in the selected lines. Overall, silencing resulted in ∼40 to 65% reduction of *TaIPK1* transcript in the seed tissue (**Figure 3B** and Supplementary Figure S5). The transgenic lines, S3-D-6-1; S6-K-3-3; S6-K-6-10 and S16-D-9-5, representing distinct events showing the significant level of silencing were therefore selected for analysis. Significant reduction of PA ∼28–56% in the mature T_4_ grains was observed when compared to the control seeds (**Figure 3C**). The maximal PA reduction was observed was for S3-D6-1 and S6-K-3-3 lines, when compared to control seeds. Overall, our data suggested that silencing of *IPK1* in wheat causes significant lowering of PA and thereby, enhancing free Pi.

### Phenotypic Changes in the Spikes of Transgenic Wheat

The germination pattern of the T_2_ seeds was studied to check any effect of lowering of PA or the integration of the plasmid. In general, no significant difference was observed for the percentage germination of seeds for the transgenic and non-transgenic control. Additionally, no delay in the germination for the transgenic seeds were observed (Supplementary Figure S6). This might suggest that the T-DNA integration does not hamper the viability of transgenic wheat seeds.

**FIGURE 4:**
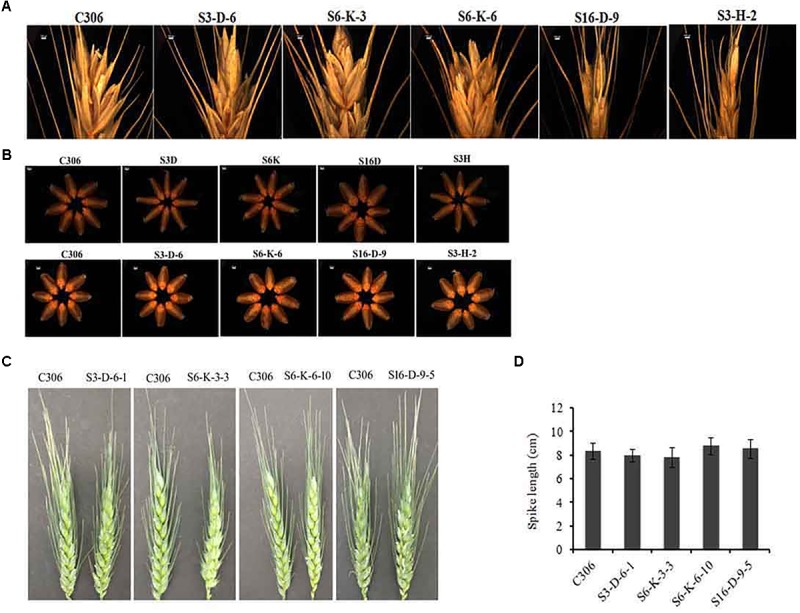
Phenotypic characteristics of *TaIPK1*:RNAi lines. **(A)** Representative pictures for grain filling at the spike head on wheat RNAi lines and control (C306) and **(B)** Representative pictures of seeds collected from wheat RNAi lines and control (C306) at T_2_ and T_3_ stages. Eight seeds were selected randomly from C306 and *TaIPK1*:RNAi lines and images were captured using a light microscope (Leica Microscope). **(C)** Representative pictures of wheat caryopsis on the onset of flowering of control C306 and *TaIPK1*:RNAi lines at T_4_. **(D)** Spike length of the primary tiller of transgenic and control plants representing T_4_ stage. Each bar indicates the mean of eight biological replicates.

**FIGURE 5:**
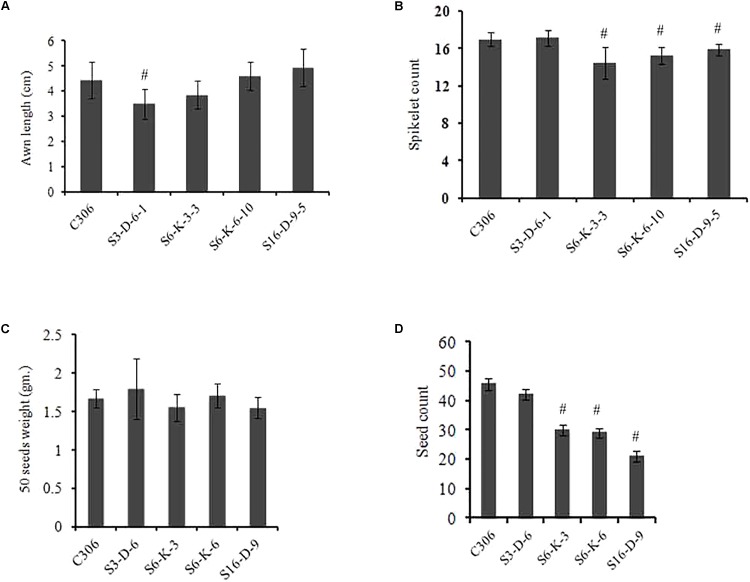
Spike characteristics of wheat transgenic plants. **(A)** Awn length from primary tiller of control C306 and *TaIPK1*:RNAi lines. **(B)** Spikelet count from the primary tiller of *TaIPK1*:RNAi lines and control C306 and at T_4_ stage. **(C)** Seed weight of control C306 and *TaIPK1*:RNAi lines. Average seed weight was measured by weighing 50 random seeds from each line. The data shown here were collected from T_3_ progenies. Each bar indicates the mean of three biological replicates (three technical replicates). **(D)** Seed count from the primary tiller of control C306 and *TaIPK1*:RNAi lines for T_3_ progenies. Each bar indicates the mean of three biological replicates. The data in **(A,B)** indicate the means of eight biological replicates. ^#^Indicates significant differences at *p* < 0.05.

The morphological traits of the selected events in T_3/4_ generation were compared with the non-transgenic plants. Multiple physiological and phenotypic parameters contributing to the grain yield (grains count per spike, spikelet count, seed weight, spike length, and awn length) were determined. Slight head sterility was observed in a few of the transgenic lines compared to non-transgenic control plants (**Figure 4A**). Transgenic wheat grains showed insignificant alteration in the grain morphology (**Figure 4B**). None of the changes in the glume arrangement or spike length were observed, suggesting the typical spikelet arrangement (**Figures 4C,D**). The awn length was found to be almost similar except for the line S3-D-6-1 as compared to non-transgenic control plants (**Figure 5A**). Developing spikes of transgenic lines did not show any significant changes in the spikelet count, except for the line S6-K-3-3, wherein a reduction of 12–14% was observed (**Figure 5B**). Seed count and seed weight were measured in the mature grains of non-transgenic wheat and *TaIPK1*:RNAi lines and observations were noted. Although, no significant differences were observed between the average seed weight of *TaIPK1*:RNAi lines and control, but had a substantial effect on total seed count (**Figures 5C,D**).

### Reduction of PA in Grains Enhances Micronutrient Content and Perturb Expression of Phosphate Transporters

**FIGURE 6:**
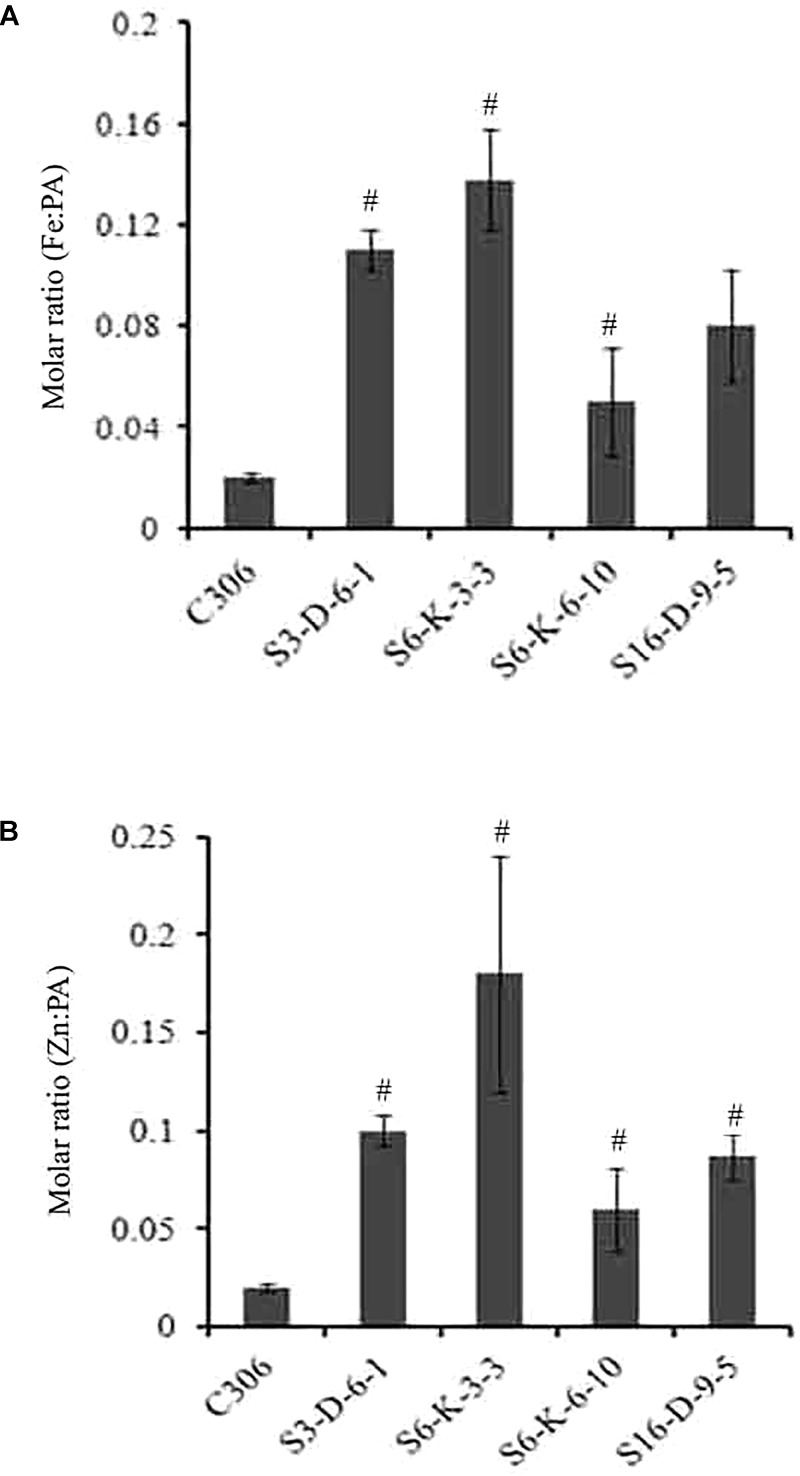
Molar ratio of Fe:PA and Zn:PA in different transgenic lines of wheat. **(A,B)** Molar ratios of Fe:PA and Zn:PA was calculated of four different transgenic wheat lines and were compared to the control (C306) seeds. The standard bar indicates average of four technical plants for each of the respective lines. Mean values showed a significant difference at *p* < 0.05 (#) with respect to their control.

In order to check if wheat *IPK1* transgenic RNAi lines showed any altered accumulation of micronutrients, transgenic seeds were analyzed for metal content. Analysis was performed using ICP-MS that suggested a significant increase in the accumulation of Zn and Fe in grains of multiple transgenic lines of T_3_ as compared to non-transgenic seeds (Supplementary Figure S7). Although, the Fe content was variable among the transgenic events, the accumulation of Zn was consistently high. The results showed ∼1.2- to 1.7-fold increase in the levels of Fe and ∼1.3- to 2.2-fold increase in Zn. Maximum accumulation of Fe and Zn was observed in S6-K-3, S6-K-6, and S16-D-9 lines. Similar observation was recorded for the T_4_ grains, where increased accumulation of Fe and Zn was observed (**Table 1**). Overall, this data suggested that lowering of PA in wheat, mediated by silencing of *IPK1* resulted in enhanced accumulation of Zn and Fe. Increased molar ratio of Zn/PA and Fe/PA was observed in multiple lines that negatively correlated with PA levels in wheat grain (**Figures 6A,B**). The improved metal to PA molar ratio was consistent in both T_3_ and T_4_ generation seeds.

**Table 1:** Metal concentration of Fe and Zn in the T_4_ mature grains of wheat transgenic and non-transgenic control (C306).

Transgenic lines	Fe (μg/g)	Zn (μg/g)
C306 (Control)	45.76 ± 3.2	34.56 ± 3.4
S3-D-6-1	71.09 ± 2.1	67.03 ± 3.5
S6-K-3-3	80.09 ± 8.1	50.71 ± 6.7
S6-K-6-10	60.49 ± 4.1	51.78 ± 4.7
S16-D-9-5	72.45 ± 3.4	55.71 ± 6.7

*The standard deviation was calculated from the data obtained from the grains pooled from at least three to four individual plants (mid portion of primary spike) of the T_*3*_ lines representing the subsequent lines.*

Since, an increase in the free Pi was observed, we also studied the expression of previously identified phosphate transporter (PHT1 sub-family) genes in the transgenic lines (Shukla et al., 2016). Many of the wheat PHTs were differentially expressed in these transgenic lines suggesting that lowering of PA impacts their transcript levels (Supplementary Figure S8). Taken together, our data conclude that changes in the content of Pi in the grain tissue impacts expression pattern of PHTs.

## Discussion

The present investigation deciphers functional importance of IPK1 for its role in the accumulation of PA in hexaploid wheat. *IPK1* had been a target of choice to achieve low phytate crops (Stevenson-Paulik et al., 2005; Shukla et al., 2009; Ali et al., 2013a). Our study also demonstrated that lowering of PA in hexaploid wheat could result in enhanced micronutrient accumulation of Zn and Fe. Therefore, this study indicates that *IPK1* is a good candidate of wheat to alter PA content and thereby enhancing micronutrient bioavailability.

### *IPK1* as a Candidate Gene for Developing Low Phytate Crops

Earlier, a strong correlation was observed between expression of *IPK1* and PA accumulation during the early phase of wheat grain development (7–28 DAA) (Bhati et al., 2014). This reinforces that, the IPK1 enzyme plays important role in the PA biosynthesis during grain maturation. Subsequently, current study observed that all the three homoeologs of IPK1 were expressed in all seed tissues including vegetative parts (**Figure 1** and Supplementary Figure S2). The functionality of *IPK1* gene was demonstrated in multiple crops by enzymatic characterization or by utilizing multiple yeast mutants to rescue their defective growth phenotypes (Sweetman et al., 2006; Sun et al., 2007; Suzuki et al., 2007). Wheat *IPK1* could rescue *Sc*Δ*ipk1* mutant thermo-sensitivity and therefore confirms its active function in the heterologous system (Bhati et al., 2014). Moreover, TaIPK1 also had 83.5 and 53.2% similarity to OsIPK1 and AtIPK1 reinforcing the suitability of this candidate gene for developing *lpa* wheat. Therefore, it could be speculated that like rice, wheat *IPK1* is a suitable candidate for utilizing recent biotechnological tool like genome editing. Our further investigation led to the identification of another *IPK1*-like gene (TRIAE_CS42_4BL_TGACv1_320862_AA1050490.1) located on chromosome 4. The 270 aa sequence lacks the prerequisite domains required for a functional kinase activity (González et al., 2010). Therefore, it is possible that the identified new wheat *IPK1* lacks kinase activity. In this study, to overcome the limitation of functional redundancy in hexaploid wheat, conserved region from three homoeologs of *TaIPK1* was targeted. Subsequently, a significant decrease in the transcript of *IPK1* (40–65%) was achieved. In addition to that, reduction in mRNA *of TaIPK1* homoeologous was also observed in these transgenics (Supplementary Figure S5B). This emplies that targeting the conserved region of the sequences in wheat is an excellent strategy for functional validation studies (Gasparis et al., 2011; Bhati et al., 2016).

During this study, transgenic wheat lines with low PA show increased Pi content that is typical for *lpa2* phenotype. Previously, *lpa2* phenotype was observed in rice, maize, *Arabidopsis*, and soybeans (Raboy et al., 2000; Shi et al., 2003; Stevenson-Paulik et al., 2005; Yuan et al., 2012; Ali et al., 2013a). A summary of such approaches to reduce PA has been reflected in Supplementary Table S2. In our case, transgenic lines showed increased Pi (upto 1.6-fold) as compared to control plants. Likewise, *Arabidopsis* and soybean *ipk1* mutant showed increase of free Pi (Stevenson-Paulik et al., 2005; Yuan et al., 2012). In wheat, 56% reduction of PA was observed, as compared to 32% decrease during the earlier study (Bhati et al., 2016). This suggests that *IPK1* is a valuable candidate to significantly reduce PA in wheat grains. Although, candidate genes like *MIPS, ITPKs* are yet to be tested in wheat, but IPK1 is certainly one of the promising candidate for developing *lpa* trait. In another study mutagenized M2 wheat lines caused 37% reduction in PA content in seeds (Guttieri et al., 2004). However, *lpa* mutants of barley and maize showed approximately 50–95% reduction in PA (Raboy et al., 2000; Dorsch et al., 2003). Thus, one could speculate that complex hexaploid wheat genome confers a greater buffering capacity as compared to diploid genomes of barley and maize. Additionally, one could measure the enzymatic activity of wheat IPK1 arising from multiple genomes, i.e., A, B, and D.

### IPK1 Function Is Conserved across Species

Earlier studies showed that lowering of PA is often associated with some pleiotropic effects (Guttieri et al., 2004; Stevenson-Paulik et al., 2005; Bhati et al., 2016). Therefore, comprehensive analysis was performed for various morphological traits such as seed weight, seed count, and germination ability. Silencing of *IPK1* in rice did not show any negative effects during seed germination or other agronomic traits (Ali et al., 2013a). In contrast, our data indicated that lowering of PA by targeting *TaIPK1* impacts seed count (**Figure 5D**). The transgenic wheat in our study did not show any pleiotropic changes for seed weight and germination (**Figure 5** and Supplementary Figure S6). Taken together, this study and previous support validate the possibility that plant IPK1 as an important candidate gene for developing reduced-phytate trait in multiple crop species (Shi et al., 2007; Shukla et al., 2009; Ali et al., 2013a).

Very limited studies have been performed to study the changes in global gene expression for *lpa* mutants during seed development. Earlier, it was shown that *lpa* mutations effects global gene expression pattern for those involved in apoptosis, inositol phosphate synthesis, oligopeptide, and transmembrane transporters (Redekar et al., 2015; Zhang et al., 2016). Our study suggested that over-accumulation of Pi in seeds could cause differential changes in the wheat PHTs. In *Arabidopsis*, IPK1 were linked to a phosphate sensing and phytate production in seeds (Stevenson-Paulik et al., 2005). Most of the wheat transgenic lines obtained in this study showed high total phosphorus (Supplementary Figure S7). Herein, our data confirmed that silencing of *IPK1* not only resulted in increased free Pi but also impact the expression of wheat PHTs and thereby impacting the dynamics of Pi transporter in *lpa* wheat grains.

From previous studies, it is evident that the PA had a high affinity for micronutrients such as Zn, Fe, Ca, etc. (Vohra et al., 1965; Persson et al., 1998; Iwai et al., 2012) and renders these minerals unavailable for gut absorption. It seems that unless significant lowering of PA is achieved, no effective changes in the seed micronutrient content will be observed (Bhati et al., 2016). In the current study, reduced wheat PA content (upto 56%) in grains exhibited a concomitant increase of Fe and Zn levels (**Figures 3C**, **6**). It signifies that it is necessary to target genes that could potentially suppress PA biosynthesis efficiently. In another study wheat lines generated achieved Fe and Zn as high as 2.1- and 3.7-fold by overexpressing *OsNAS2* (Singh et al., 2017). Enhanced micronutrient levels do not correspond to metal bioavailability. However, the metal to PA molar ratios could be an indirect evidence for enhanced bioavailability (Morris and Ellis, 1989; Ma et al., 2005). Our data showed an increase in the Fe:PA and Zn:PA molar ratios. This suggests that lowering of PA enhances micronutrient molar ratio, and thus, possibly increasing the mineral bioavailability. Similarly, lowering of PA in wheat-*Aegilops* derivatives and *Triticum monococcum* enhances the bioavailability of zinc (Salunke et al., 2012). The strong validity of the observed bioavailability of micronutrients with the phytate:mineral molar ratios in the *lpa* mutants reinforce the significance of utilizing these parameters for the micronutrient biofortification programs.

This study is an important step to achieve high micronutrients in grains of the hexaploid wheat. Recently researchers have also focused on understanding Zn and Fe transport as a novel route to improved loading in developing grains (Rouached, 2013; Grillet et al., 2014). Such parallel approaches for enhancing micronutrients have been recently evaluated by overexpressing certain metal transporters, phytases, etc (Abid et al., 2017; Connorton et al., 2017; Singh et al., 2017). Altogether, such strategies reinforce the importance of achieving enhanced bioavailable Fe and Zn in cereal crops to address malnutrition.

## Author Contributions

AP conceived the idea and designed the experiments. SA performed most of the experiment. KB, AK, and ST helped in the experiments dealing with wheat transformation. VS and GK performed the experiments related to transporters. AP, SA, KB, and ST wrote the article. All the authors read and approved the manuscript.

## Conflict of Interest Statement

The authors declare that the research was conducted in the absence of any commercial or financial relationships that could be construed as a potential conflict of interest.

## Abbreviations

ABC-MRP: ATP binding cassette multi drug resistance protein
DAA: day after anthesis
lpa: low phytic acid
PA: phytic acid.
