# Supplementary material for: RNAi-Mediated Downregulation of Inositol Pentakisphosphate Kinase (IPK1) in Wheat Grains Decreases Phytic Acid Levels and Increases Fe and Zn Accumulation
Source: Front Plant Sci. 2018 Mar 6;9:259. doi: 10.3389/fpls.2018.00259 (PMC5845732; doi:10.3389/fpls.2018.00259)
Supplement: Supplementary file 1 [file Table_1.DOCX]

**Supplementary Table S1** Primers used in the current study.

| **Name of Gene** | **Primers (5**'**-3**'**)** |
| --- | --- |
| *TaIPK1:2A* | F: TGCTGCATGCCGGCGACGCCGA  R: TACGCAGTTTGGTGCTGGTTGAGCC |
| *TaIPK1:2B* | F: GGGGCGATTCATCTGTATTACGACATC  R: CGCGGTAGCAGAAATAAGAAAGTCCCTGACT |
| *TaIPK1:2D* | F: ACCGGCAACTCGCCTTCCATGTTT  R: TGACATGTACTGCATAGGCTTGTGCC |
| *TaIPK1*  *(RNAi insert)* | F: ACTAGTGGCGCGCCCATGAGCCAACATCTGGGGCA  *(SpeI/AscI)*  R: GCGATCGCCCTAGGTGAGGTGTTGATGCATCTTGTACC *(SgfI/AvrII)* |
| *bar* | F: AGATCTCGGTGACGGGCAGGA  R: CGACATCCGCCGTGCCACCGAG |
| *OCS1* Terminator | F: CGAGCGGCGAACTAATAACG  R: AATTCTCGGGGCAGCAAGTC |
| *TaIPK1* | F: GCTCGTGTGGGGTGACATCCCAG  R: CAGGACGGGCGCTGAGCACAT |
| *TaYSL1(qPCR)* | F: CCCTGTCCTCATCAACTTCTCCATGCTTTTCG  R: GGCAGCCCTTATGGTGATTGAACCCAGTTG |
| *TaYSL2(qPCR)* | F: GGCGCGGGGATGATCTGCTCACACC  R: GGCCATCCCCCATGATCAGAGCTACACAC |
| *TaYSL12(qPCR)* | F: GAAGAAGAACAGCACCATCCCGGTCTCG  R: ATGTAGTACCACTTGAGGTCCGGGAAGATC |
| *TaYSL14(qPCR)* | F: CCAGAATCTAGTCTTCATGGGCTGCAGGC  R: GCTCATCATCAAAGGACATAGCTTCAGCGG |
| *TaYSL15(qPCR)* | F: ATGACATACGTTGGTGCCGGGATGATTTGCCC  R: CCCCCATGATGAGAGCTATGCATATGAAGGCC |
| *TaYSL15-Like(qPCR)* | F: AGCTTGCTGGACCGCTTCGGCATCGTG  R: CCAATCCCTGGCTCCTTGTAGCTCCCC |
| *TaYSL16(qPCR)* | F: CGGGTTCTGCAACGCCTACGGCAC  R: ACGTCAGCGTCAGGTGCGCCGTC |
