# Supplementary material for: RNAi-Mediated Downregulation of Inositol Pentakisphosphate Kinase (IPK1) in Wheat Grains Decreases Phytic Acid Levels and Increases Fe and Zn Accumulation
Source: Front Plant Sci. 2018 Mar 6;9:259. doi: 10.3389/fpls.2018.00259 (PMC5845732; doi:10.3389/fpls.2018.00259)
Supplement: Supplementary file 2 [file Table_2.docx]

**Supplementary Table S2** List of crops where *lpa2* type phenotype was observed and subsequently it was mapped or co-related with the functional activity of IPK1. Corresponding phenotypes observed in these lines were also mentioned.

| ***IPK1* orthologs** | **Functional approaches** | **Phenotype** | **References** |
| --- | --- | --- | --- |
| *AtIPK1*  (*Arabidopsis thaliana*) | T-DNA insertion mutants | Low PA, Pi sensing, Root hair elongation | (Kuo et al., 2014; Stevenson-Paulik et al., 2005) |
| *ZmIPK1*  (*Zea mays*) | Zinc-finger nucleases | Low PA, High Pi | (Shukla et al., 2009) |
| *OsIPK1*  (*Oryzae sativa*) | RNAi (seed specific promoter) | Low PA, High Pi | (Ali et al., 2013) |
| *GmIPK1*  (*Glycine max*) | Physical mutagenesis  (Gamma irradiation) | Low PA, High Pi | (Yuan et al., 2007, 2012) |
| *TaIPK1*  *(Triticum aestivum)* | RNAi | Low PA, High Pi | This study |
