# Supplementary material for: RNAi-Mediated Downregulation of Inositol Pentakisphosphate Kinase (IPK1) in Wheat Grains Decreases Phytic Acid Levels and Increases Fe and Zn Accumulation
Source: Front Plant Sci. 2018 Mar 6;9:259. doi: 10.3389/fpls.2018.00259 (PMC5845732; doi:10.3389/fpls.2018.00259)
Supplement: Supplementary file 3 [file Image_1.pdf]

RNAi-mediated downregulation of inositol pentakisphosphate kinase (IPK1) in wheat grains decreases phytic acid levels and increases Fe and Zn accumulation

Sipla Aggarwal[1,2], Anil Kumar[1], Kaushal Kumar Bhati[1,3], Gazaldeep Kaur[1], Vishnu Shukla[1], Siddharth Tiwari[1] and Ajay Kumar Pandey[1*]

Author affiliations

[1]National Agri-Food Biotechnology Institute (Department of Biotechnology), Sector 81, Knowledge City, Mohali-140306, Punjab, India.

[2] Department of Biotechnology, Panjab University, Punjab, India.

[3]Copenhagen Plant Science Centre, PLEN, University of Copenhagen, Thorvaldsensvej 40, 1871 Frederiksberg C, Denmark

Corresponding author:

Dr. Ajay K Pandey, Scientist-E.
National Agri-Food Biotechnology Institute (Department of Biotechnology), Sector 81, Knowledge City, Mohali-140306, Punjab, India.
Telephone: +91-1724990124
Email: pandeyak@nabi.res.in; pandeyak1974@gmail.com

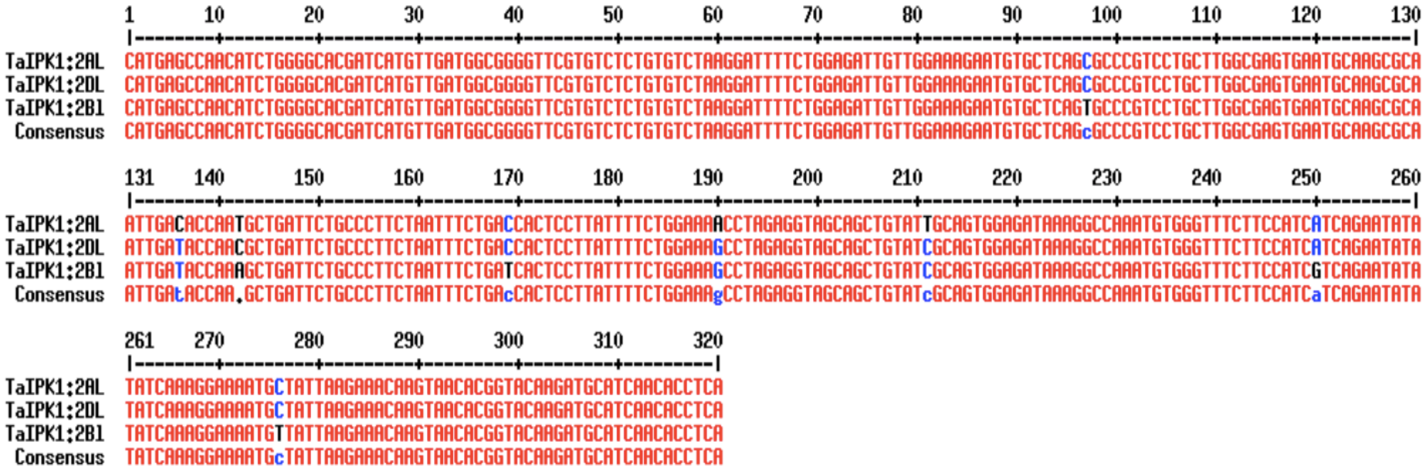

Supplementary Fig. S1 Multiple sequence alignment of *TaIPK1* transcripts used to design RNAi targets. The nucleotide sequence of transcripts were retrieved from three different homoeologues; 2AL, 2BL, 2DL and alignment was prepared using multAlin.

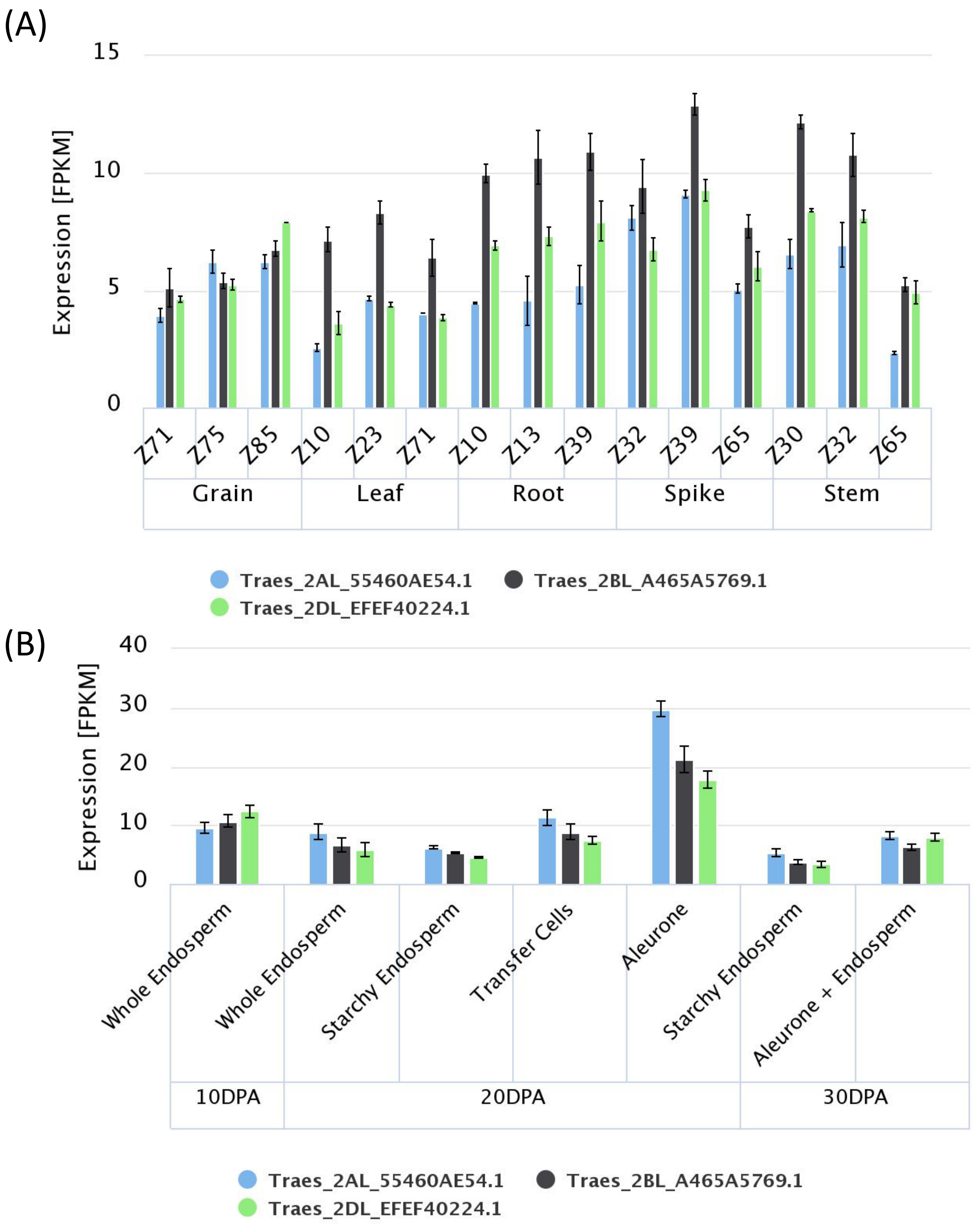

Supplementary Fig. S2 Expression pattern of homoeologs of *IPK1* gene in different organs of wheat plants. (A) Expression in five different tissues and three developmental stages from hexaploid wheat. z, Zadoks scale (Zadoks et al., 1974); (B) in different grain tissues at 10, 20 and 30 DPA (days post anthesis).

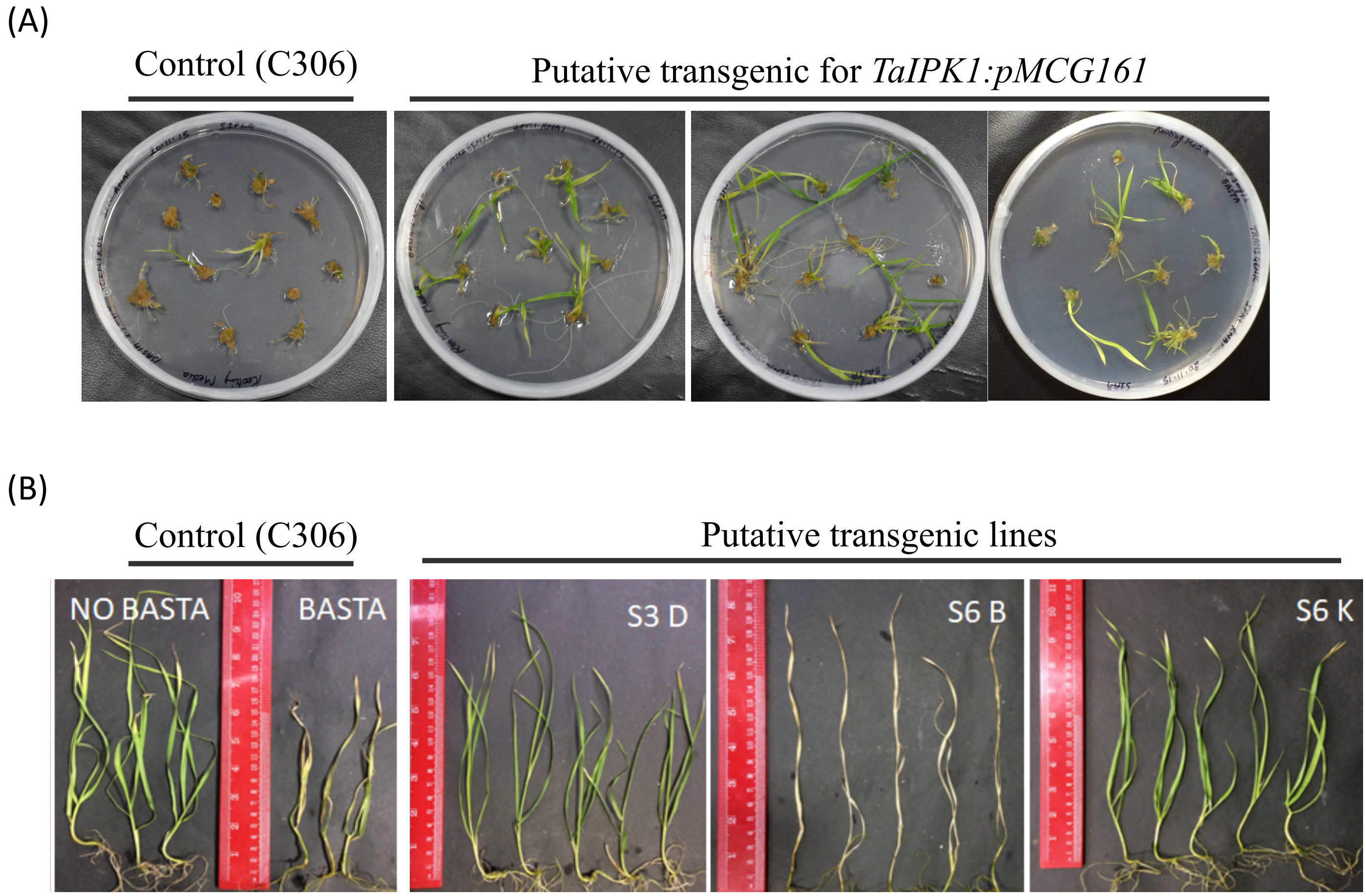

Supplementary Fig. S3 Selection of wheat RNAi transgenic plants. (A) Representative images of transgenic seedling (right panels) selected over BASTA (2.5 mg/L) w. r. t. control (left panel). (B) Representative images for the plants surviving the BASTA selection during the screening process of wheat RNAi in hydroponics system.

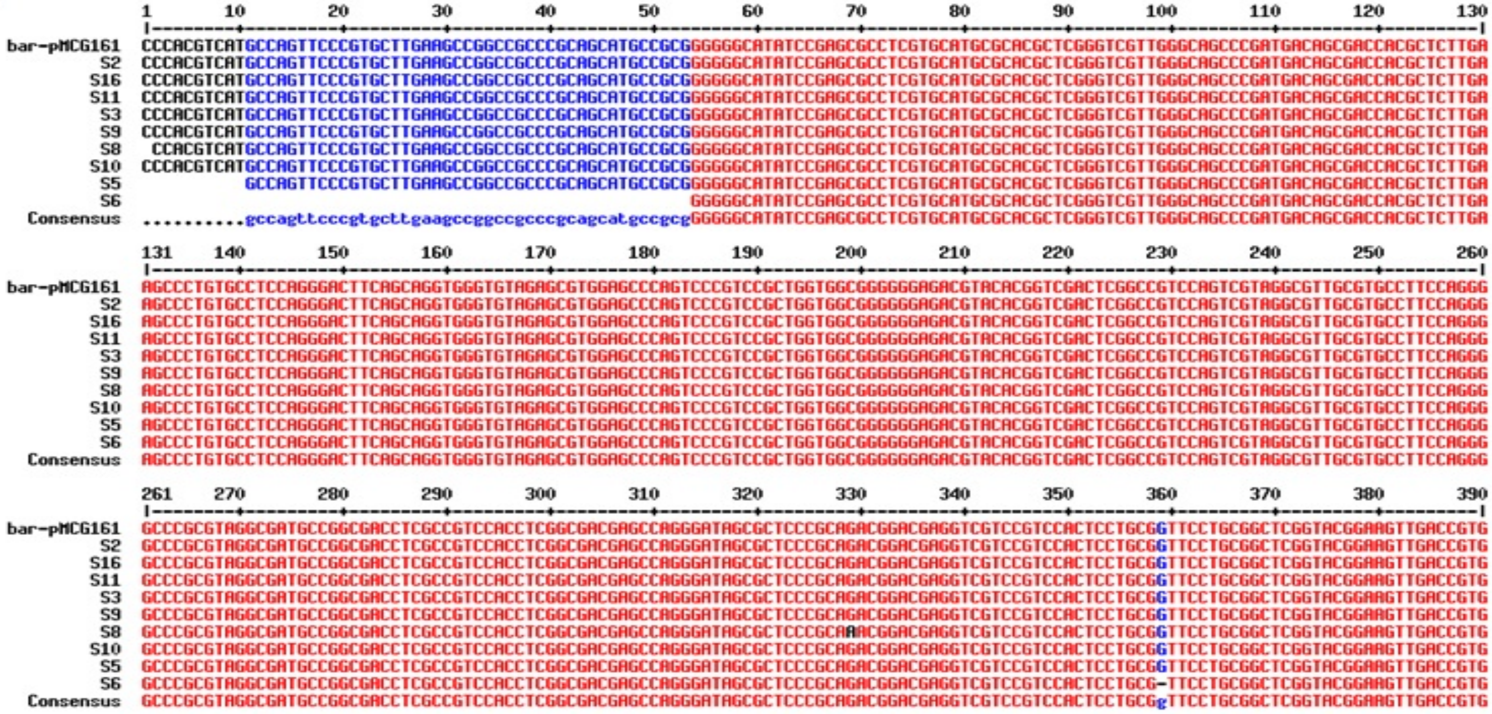

Supplementary Fig. S4 Sequence confirmation of *bar* gene amplicon from wheat transgenic plants. PCR amplicon was gel eluted, purified and sequenced for confirmation. The above is a representation of amplicon sequences obtained for T_0 events .

(A)

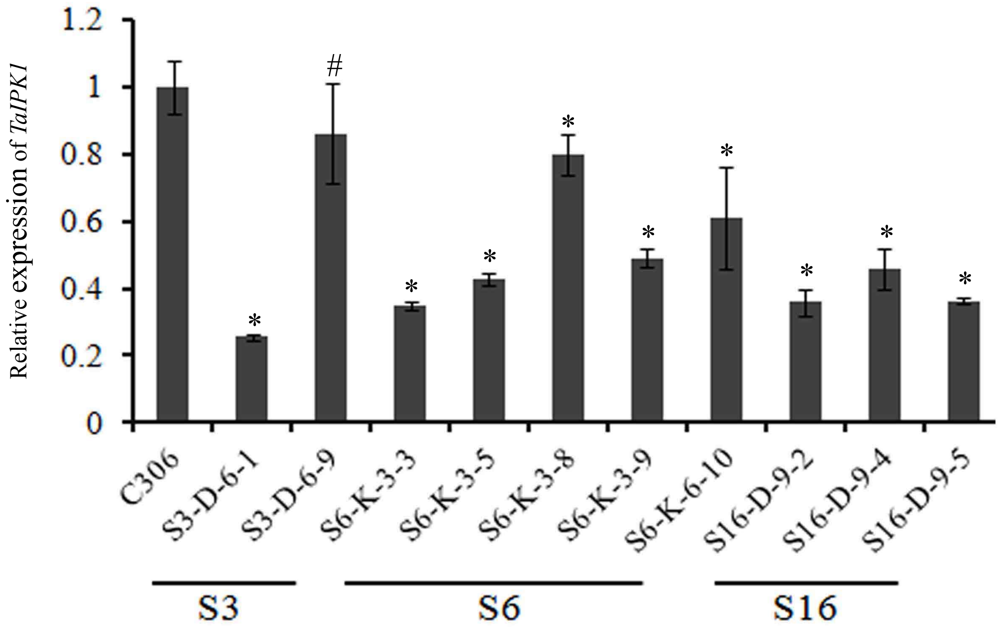

(B)

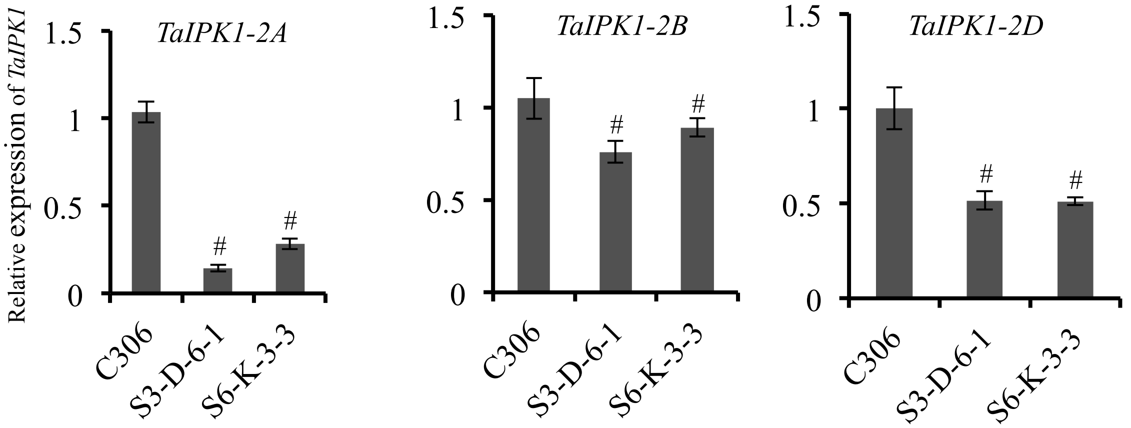

Supplementary Fig. S5 qRT-PCR analysis of *TaIPK1* transcript in transgenic and non-transgenic control lines. (A) Non segregating RNAi lines from three independent integration events were subjected to quantification of *TaIPK1* in multiple 14DAA immature seeds (T_4). (B) Homoeolog specific gene silencing of *TaIPK1* arising from A, B and D genome was performed on the selected lines. Two µg of DNA free total RNA was used for cDNA preparation. Real time assays were performed using SYBR Green and target gene Ct values were normalized using wheat *ARF* gene as internal control. Results indicate means ± SD with three to four technical replicates. # and * indicates significant difference at $p<0.05$ and $p<0.01$ with respect to C306.

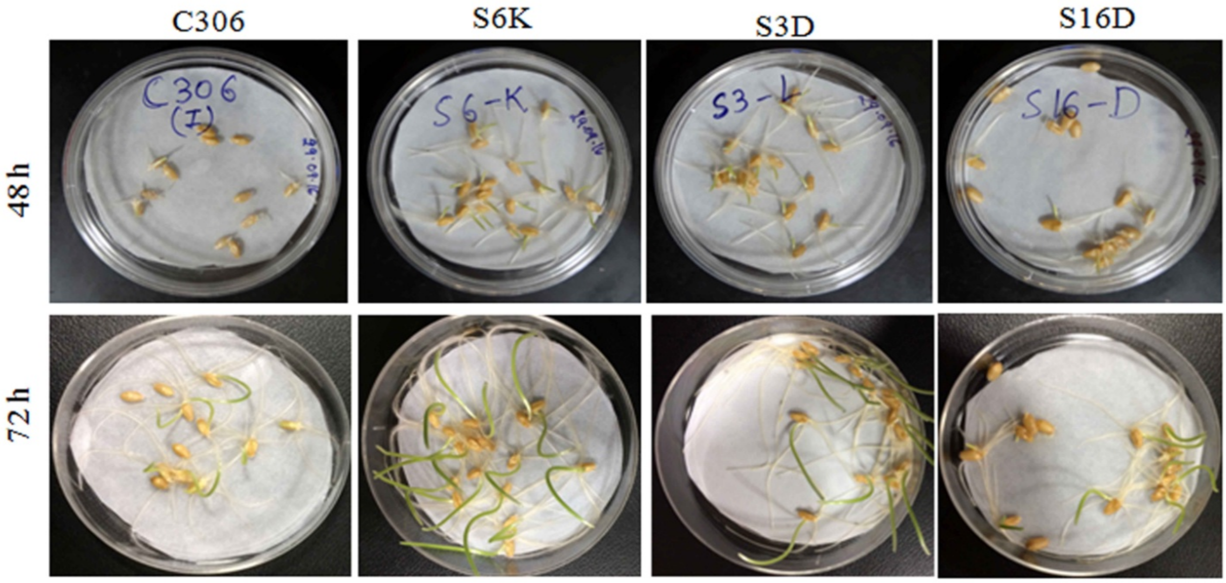

Supplementary Fig. S6 Representative picture of T_2 transgenic seeds showing the germination pattern with respect to non-transgenic. Mature seeds (12-16 grains) from each transgenic line and control C306 plants were collected, sterilized and then kept for germination. Pictures were taken after 48 hr and 72 hr post germination.

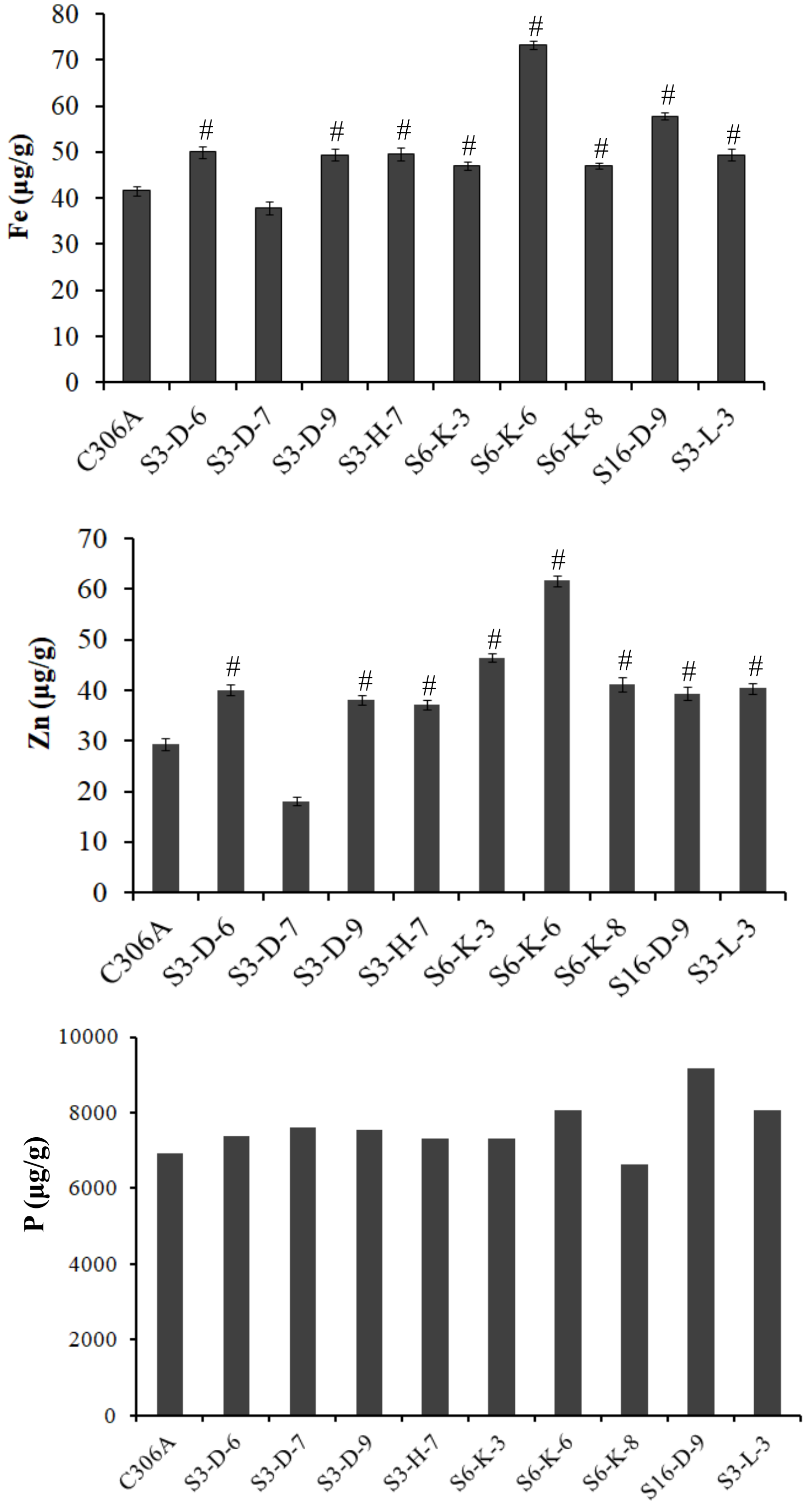

Supplementary Fig. S7 ICP-MS analysis of Fe(µg/g), Zn (µg/g) and P (µg/g). Metal analysis of the mature grains from the individual plants (mature seeds of wheat transgenic at T_3 stage). Analysis was done as mentioned in experimental procedure, by using ICP-MS. Standard deviation indicates the mean of four technical replicates. (# indicates significant differences at $p<0.05$ with respect to C306)

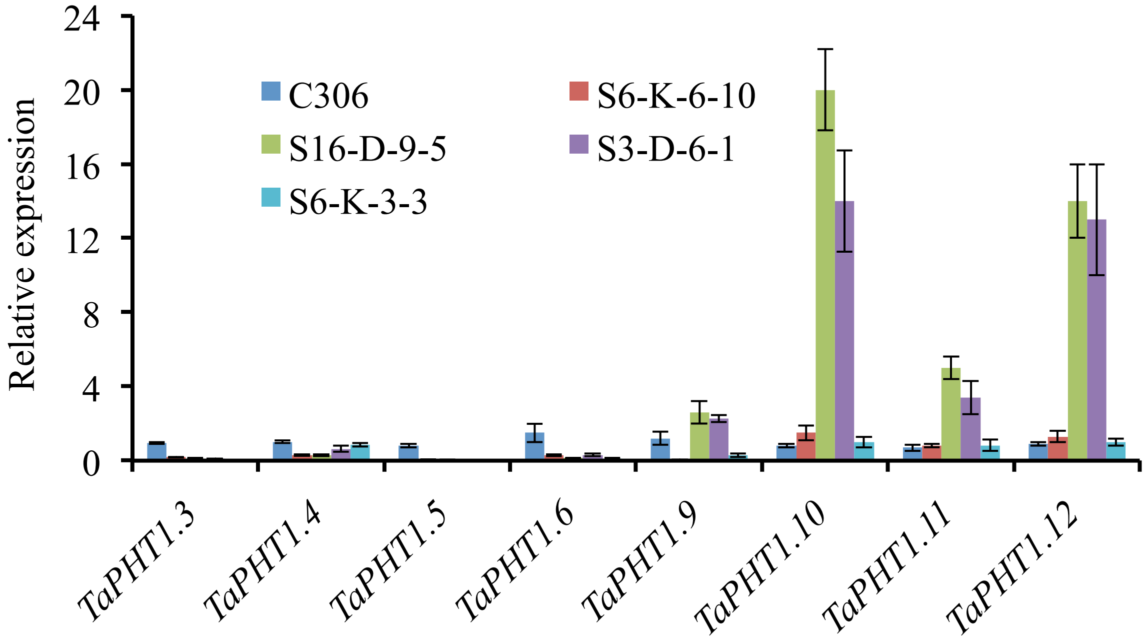

Supplementary Fig. S8 qRT-PCR analysis of wheat phosphate transporters (PHTs) in seeds of the transgenic lines (S3-D-6-1, S6-K-3-3, S6-K-6-10 and S16-D-9-5) and non-transgenic control (C306). Two µg of DNA free total RNA was used for cDNA preparation. Real time assays were performed using SYBR Green and target gene Ct values were normalized using wheat *ARF* gene as internal control. Fold level of each PHT gene in transgenic was calculated with respect to the their expression in control non-transgenic C306. Results indicate means ± SD with three technical replicates.
